# From microbiome dysbiosis to virtual reality and nanomedicine: a multidisciplinary roadmap for pediatric glaucoma care

**DOI:** 10.1097/MS9.0000000000004154

**Published:** 2025-10-17

**Authors:** Muhammad Talha, Muhammad Bilal, Mahnoor Fatima, Muammar Hassan, Omar Abdullah Gill

**Affiliations:** a Department of Medicine, King Edward Medical University, Lahore, Pakistan; b Department of Medicine, Shaheed Ziaur Rahman Medical College, Rajshahi Medical University, Bangladesh

*To the Editor,*

Pediatric glaucoma is a rare, complicated group of disorders characterized by elevated intraocular pressure (IOP) resulting in progressive optic neuropathy and severe vision impairment during critical developmental intervals. Recently, findings have proposed that the gut-eye axis microbiome is an important regulator for ocular homeostasis, with dysbiosis inciting neuroinflammation and glaucomatous neurodegeneration. Recent developments suggest that nanomedicine particles as new agents capable of precisely modulating such microbiomes to restore immune balance for possibly delaying glaucoma development, especially in children who are implanted with neurotechnology devices^[1,2]^. This is in line with the Transparency In The reporting of Artificial INtelligence (TITAN) Guidelines on the need for transparency in AI use in healthcare^[3]^.

Microbiome changes affecting IOP in syndromic pediatric glaucoma continue to have a clinical approach exhibiting much variability. Virtual reality (VR)-based visual field simulation is becoming important in caring for pediatric glaucoma by providing an engaging assessment of visual function for children, and one that is superior to traditional methods. Predicting and simulating IOP fluctuations due to microbiome effects using VR is still rather undeveloped, particularly since recent work has indicated that the gut-eye microbiome may affect ocular physiology via metabolites such as short-chain fatty acids. Having VR simulation platforms coupled with an understanding of the microbiome would allow for predictive modeling of treatment and dynamic outcome monitoring^[4,5]^.

Pediatric glaucoma with concurrent fetal alcohol spectrum disorders (FASD), a neurodevelopmental condition furthering the ocular insult, presents a complicated situation. Artificial intelligence (AI) applied to genomic datasets has revealed novel biomarkers, including oxidative stress-related molecules and mutations in genes such as THBS1 concerning optic nerve integrity. AI-assisted biomarker discovery takes precision medicine into overdrive to facilitate early diagnosis and tailor intervention for patients with these two conditions^[6,7]^.

Enhanced 3 Dimensional (3D) bioprinted composites by nanomedical therapy may be an intriguing opportunity for sustained microbiome adjustment or ocular tissue rehabilitation. In addition to healing tissues, these composites target pathogenic microbes by releasing antimicrobial nanoparticles like titanium dioxide, zinc oxide, and copper. Conventional approaches fail to control developmental neurotoxic deficits such as infantile glaucoma and its syndromic manifestations, which might be successfully mitigated by 3D nanomedical therapy^[2,8]^.

Recent research studies have indicated the significant presence of gram-negative bacteria in the altered ocular surface microbiome of glaucomatous patients. This is a potentially relevant factor in pediatric forms as well. A concerted approach involving gut microbiome modulation along with dysbiosis correction may improve disease control. For disease management, a holistic approach enacted through diet and lifestyle interventions, which supplement VR-enabled strategies and nanomedicine^[1,9]^.

New evidence from recent research studies on pediatric glaucoma has highlighted its potential pathogenesis in the context of microbiome dysbiosis, advanced technologies, and novel therapeutics. Perhaps encouraging advances with nanomedicine-based microbiome modulation, VR-assisted visual field assessments, and AI-groomed biomarker discovery have emerged, but most of the current evidence is still largely exploratory, fragmented, and deficient in its clinical translation. Bridging the prevalent methodological gaps, problems related to standardization, and large-scale validation deficiency is crucial for transitioning these innovations into reliable healthcare strategies, and viewing pediatric glaucoma from this multidisciplinary perspective deepens the literature by linking varied experimental findings with their translational hypothetical potential in real pediatric care^[4]^.

In conclusion, combining VR simulation technologies, AI-driven biomarker discovery, and nanomedicine for microbiome modulation offers a variety of approaches to the treatment of pediatric glaucoma. To validate these integrative approaches, rigorous, multidisciplinary research and clinical trials are desperately needed. In children with rare glaucoma variants complicated by neurotechnology implants and syndromic conditions like FASD, such advancements hold promise for earlier detection, customized treatments, and improved neurodevelopmental outcomes.

## Data Availability

Not applicable to this study.

## Research registration unique identifying number (UIN)

No human subjects were involved, as the type of research being submitted is an editorial.
